# Germline regulation of the intestinal mitochondrial unfolded protein response

**DOI:** 10.1007/s11357-025-01890-5

**Published:** 2025-11-07

**Authors:** Anna C. Foulger, Nathaniel A. Jordan, Alyssa Castle, Dipa Bhaumik, Julie K. Andersen, Gordon J. Lithgow, Suzanne Angeli

**Affiliations:** 1 Buck Institute for Research On Aging, Novato, CA 94945 USA; 2 University of Maine, Orono, ME 04469 USA

**Keywords:** Mitochondrial unfolded protein response, Germline, Cell non-autonomous, Fertilization, Reproduction, Intestines, *Caenorhabditis elegans*

## Abstract

The disposable soma theory posits that there is a trade-off between reproduction and somatic maintenance. In support of this theory, we previously identified that pharmacological inhibition of the germline has widespread protective cell non-autonomous effects on cellular protein homeostasis in the model organism *Caenorhabditis elegans*. However, the cell non-autonomous effects of the germline on mitochondrial protein homeostasis are not well defined. Here, we use pharmacological or genetic inhibition of the germline to determine its effects on intestinal mitochondrial protein homeostasis as measured by the mitochondrial unfolded protein response (UPR^mt^). We find that pharmacological inhibition of germline proliferation by 5-fluoro-2-deoxyuridine (FUdR), a DNA synthesis inhibitor, potently inhibits activation of the intestinal UPR^mt^ as well as reverses lifespan effects induced by mitochondrial dysfunction. We find similar results with the genetic mutant (*glp-1*), which lacks germline proliferation. To further identify the reproductive processes required to regulate the intestinal UPR^mt^, we examined the genetic mutant *fem-1*, which contains an intact gonad with oocytes but lacks sperm. Like *glp-1* mutants, *fem-1* mutants do not activate the intestinal UPR^mt^ due to mitochondrial dysfunction caused by loss of OXPHOS subunits. Restoring reproduction in *fem-1* mutants by mating them with wild type males is sufficient to reactivate the intestinal UPR^mt^. Furthermore, loss of the FOXO transcription factor *daf-16* is sufficient to reactivate the intestinal UPR^mt^ in *fem-1* mutants and partially in *glp-1* mutants. These findings suggest that FOXO/*daf-16* acts to limit UPR^mt^ activation in the intestine. These findings also suggest that late-stage reproductive signals that include the maturation of oocytes and fertilization may play a critical role in cell non-autonomous intestinal UPR^mt^ activation.

## Introduction

During larval development, *Caenorhabditis elegans* germline stem cells first produce sperm and then oocytes to create a hermaphroditic germline capable of self-fertilization. All tissues are post-mitotic in *C. elegans* during adulthood except for the germline. During adulthood, the sperm fertilize the oocytes until all the sperm are depleted, which typically yields approximately 300 viable progeny. The DNA synthesis inhibitor, 5-fluoro-2-deoxyuridine (FUdR), is commonly used to inhibit reproduction in adult *C. elegans* to avoid progeny contamination. In addition to inhibiting progeny development, we and others have previously demonstrated that FUdR treatment impacts cytosolic protein homeostasis in the somatic tissues of *C. elegans* [1–3]. FUdR treatment leads to the activation of the heat shock response (HSR) as demonstrated by elevation of heat shock chaperones and protection from heat stress and can protect against protein misfolding in body-wall muscles, including in a model of Alzheimer’s disease (AD) [1–3]. These findings demonstrate that pharmacological inhibition of the germline can have widespread cell non-autonomous effects on cytosolic protein homeostasis.

**Fig. 1:**
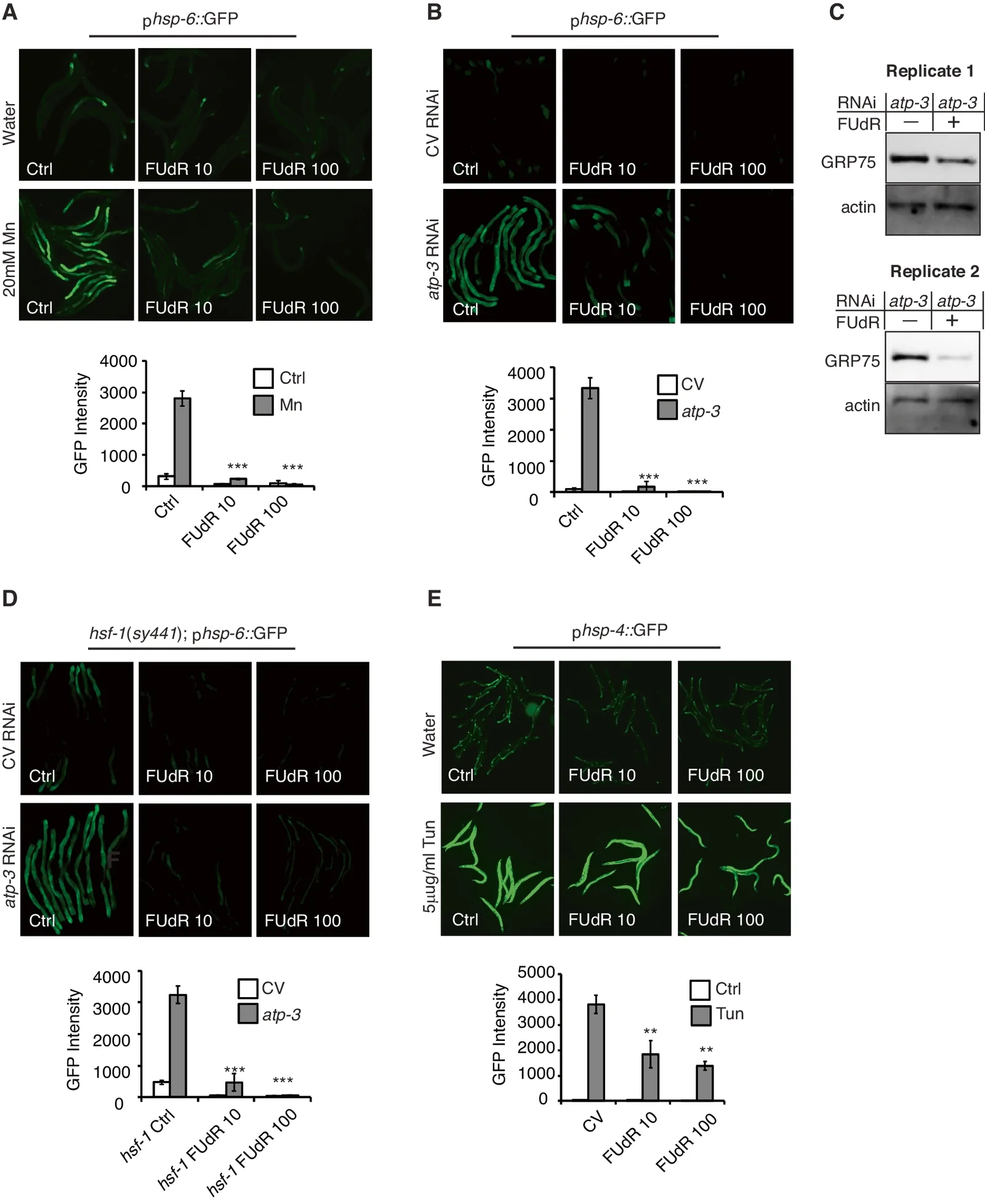
Pharmacological inhibition of the germline blocks activation of the intestinal UPR^mt^. **A. **Top panels: Photomicrographs of p*hsp-6*::GFP reporter strain after treatment with 20mM MnCl_2_ in the absence or presence of 10 or 100μg/ml FUdR. Nematodes were exposed to treatments beginning at the L4 stage for 48 hours. Bottom panel: Quantification of GFP intensity in the absence or presence of 10μg/ml or 100μg/ml FUdR and 20mM MnCl_2_. Data are the mean ± SEM of ≥ 45 animals combined from three biological experiments. ***p ≤ 0.0001 by Student’s *t*-test. **B**, **D. **Top panels: Photomicrographs of p*hsp-6*::GFP reporter strain (**B**) or *hsf-1*(*sy441*); p*hsp-6*::GFP reporter strain (**D**) after treatment with RNAi against OSCP/*atp-3* in the absence or presence of 10μg/ml or 100μg/ml FUdR. Nematodes were exposed to treatments beginning at the young adult stage for 48 hours. CV, control vector RNAi. Bottom panel: Quantification of GFP intensity. Data are the mean ± SEM of ≥ 45 animals combined from three biological experiments. ***p ≤ 0.0001 by Student’s *t*-test. **C. **Immunoblots from N2 nematodes after RNAi against OSCP/*atp-3* in the absence or presence of 100μg/ml FUdR. RNAi and FUdR were administered for 24 h beginning at the young adult stage and then collected for analysis. Representative immunoblots are from two biological experiments. Actin was used as a loading control. **E. **Top panels: Photomicrographs of p*hsp-4*::GFP reporter strain after 5μg/ml tunicamycin treatment in the absence or presence of 10μg/ml or 100μg/ml FUdR. Nematodes were exposed to tunicamycin and FUdR beginning from young adulthood for 48 hours. Bottom panel: Quantification of GFP intensity from nematodes treated with 5μg/ml tunicamycin and either 10μg/ml or 100μg/ml FUdR. Data are the mean ± SEM of ≥ 45 animals combined from three biological experiments. **p ≤ 0.001 by Student’s *t*-test

**Fig. 2:**
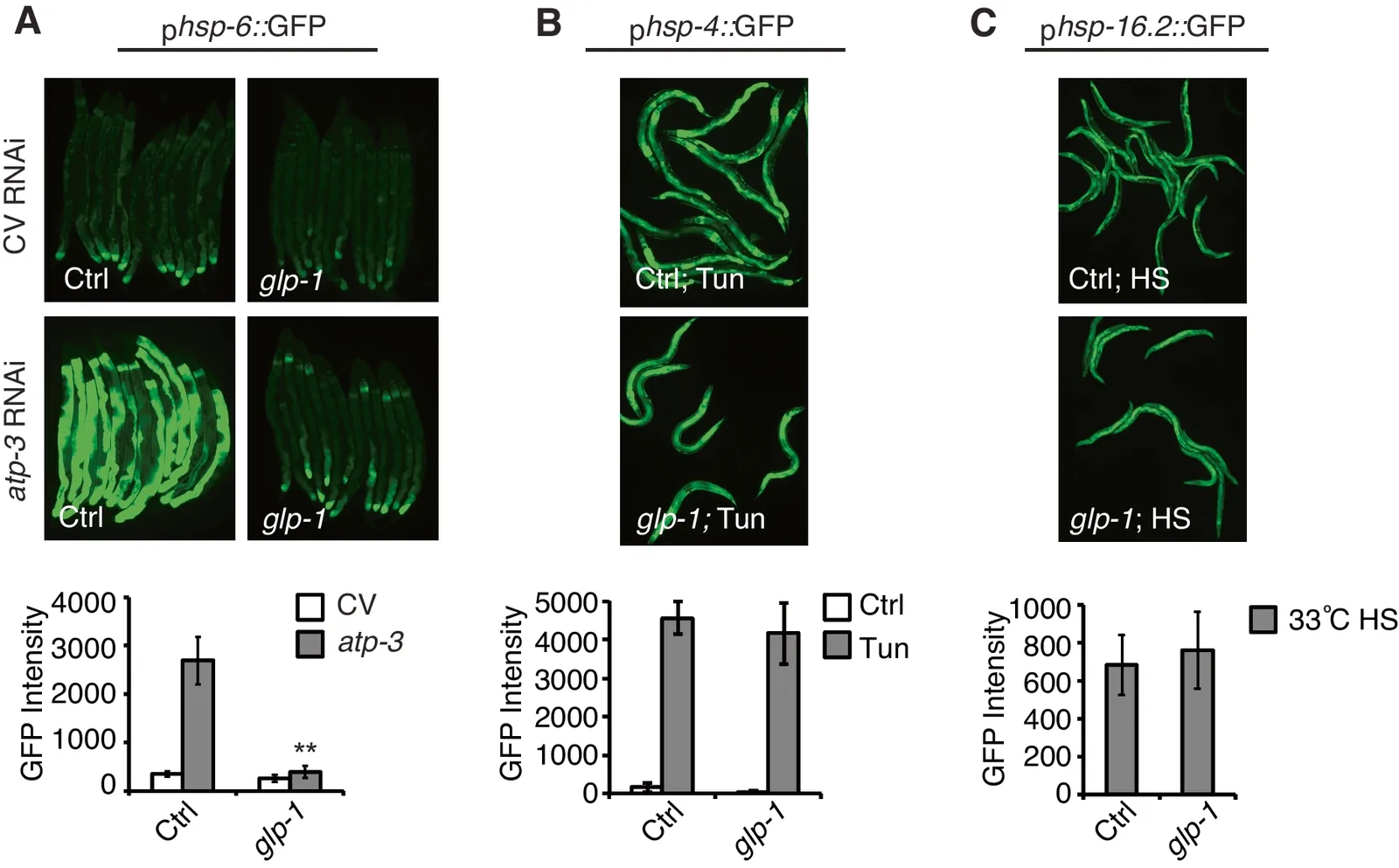
Genetic inhibition of the germline blocks activation of the intestinal UPR^mt^. **A. **Top panels: Photomicrographs of p*hsp-6*::GFP or *glp-1(e2141)*; p*hsp-6*::GFP reporter strains after treatment with bacteria expressing RNAi against OSCP/*atp-3*. Strains were developed at 25°C to induce sterility of *glp-1(e2141)* mutants. At young adulthood, nematodes were shifted to RNAi plates at 20°C for 48 hours. Bottom panel: Quantification of GFP intensity. Data are the mean ± SEM of ≥ 45 animals combined from three biological experiments. **p ≤ 0.001 by Student’s *t*-test. **B. **Top panels: Photomicrographs of p*hsp-4*::GFP or *glp-1(e2141)*; p*hsp-4*::GFP reporter strains after treatment with 5μg/ml tunicamycin. Strains were developed at 25°C to induce sterility of *glp-1*(*e2141)* mutants. At young adulthood, nematodes were shifted to tunicamycin at 20°C for 48 hours. Bottom panel: Quantification of GFP intensity. No significant difference in GFP activation was observed between p*hsp-4*::GFP or *glp-1*(*e2141*); p*hsp-4*::GFP reporter strains after tunicamycin treatment. Data are the mean ± SEM of ≥ 45 animals combined from three biological experiments. **C. **Top panels: Photomicrographs of p*hsp-16.2*::GFP or *glp-1(e2141)*; p*hsp-16.2*::GFP reporter strains after heat stress. Strains were developed at 25°C to induce sterility of *glp-1*(*e2141)* mutants. At young adulthood, nematodes were heat stressed for 1 hour at 33°C, and GFP expression was analyzed approximately 5 hours later. Bottom panel: Quantification of GFP intensity. No significant difference in GFP activation was observed between p*hsp-4*::GFP or *glp-1*(*e2141*); p*hsp-4*::GFP reporter strains after heat stress. Data are the mean ± SEM of ≥ 45 animals combined from three biological experiments

**Fig. 3:**
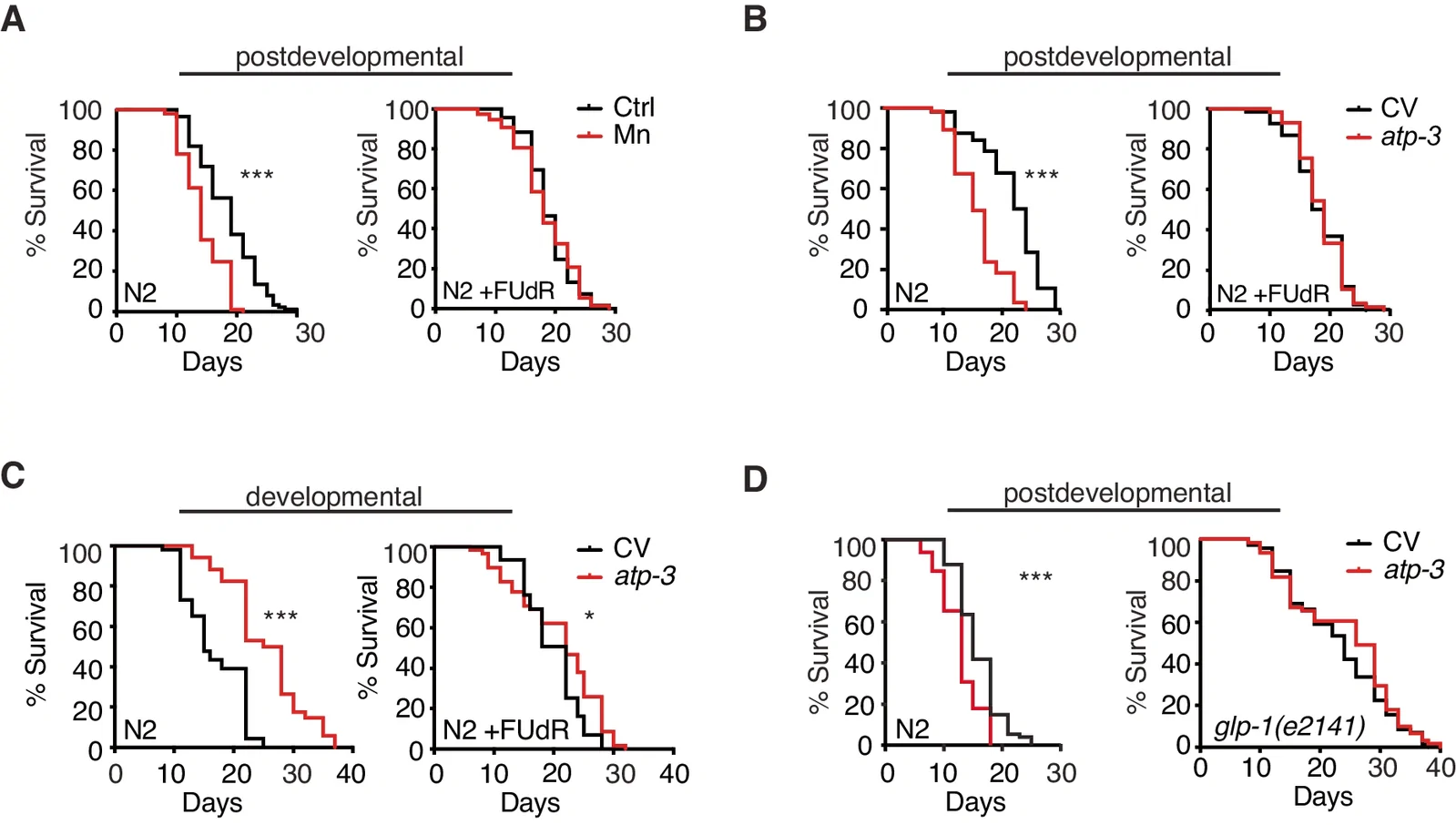
Pharmacological or genetic inhibition of the germline alters lifespans impacted by mitochondrial dysfunction. **A. **Survival curves of wild type N2s with postdevelopmental treatment of 10mM MnCl_2_ in the presence or absence of 10μg/ml FUdR. Nematodes were exposed to MnCl_2_ beginning at the L4 stage and continued throughout the entire lifespan. Representative curves were selected from three biological experiments. N2 water control (*n* = 189, median survival = 16 days), N2 10mM MnCl_2_ (*n* = 215, median survival = 14 days). ***p ≤ 0.0001 by Log Rank (Mantel–Cox) test. N2 water control, 10 μg/ml FUdR (*n* = 138, median survival = 16 days), N2 10mM MnCl_2_; 10μg/ml FUdR (*n *= 127, median survival = 16 days). *p = *0.3862 by Log Rank (Mantel–Cox) test. **B. **Survival curves of wild type N2s with postdevelopmental treatment of RNAi against OSCP/*atp-3* in the presence or absence of 10μg/ml FUdR. Nematodes were exposed to RNAi treatment beginning at the young adult stage for 48 hours and then moved to OP50 bacteria for the remainder of the lifespan. Representative curves were selected from three biological experiments. N2 CV RNAi (*n* = 108, median survival = 22 days), N2 OSCP/*atp-3* RNAi (*n* = 106, median survival = 15 days). ***p ≤ 0.0001 by Log Rank (Mantel–Cox) test. N2 CV RNAi, 10μg/ml FUdR (*n* = 120, median survival = 19 days), N2 OSCP/*atp-3* RNAi; 10μg/ml FUdR (*n* = 109, median survival = 20 days).* p* = 0.1967 by Log Rank (Mantel–Cox) test. **C.** Survival curves of wild type N2s treated with RNAi against OSCP/*atp-3* in the presence or absence of 10μg/ml FUdR. Nematodes were exposed to RNAi treatment beginning from eggs (developmental) and for their entire lifespan. Nematodes were exposed to FUdR treatment beginning from young adulthood and for the remainder of the lifespan. Representative curves were selected from three biological experiments. N2 CV RNAi (*n* = 68, median survival = 15 days), N2 OSCP/*atp-3* RNAi (*n* = 64, median survival = 26 days). ***p ≤ 0.0001 by Log Rank (Mantel–Cox) test. N2 CV RNAi, 10μg/ml FUdR (*n* = 67, median survival = 22 days), N2 OSCP/*atp-3* RNAi; 10μg/ml FUdR (*n* = 64, median survival = 22 days). *p ≤ 0.05; *p* = 0.0466 by Log Rank (Mantel–Cox) test. **D. **Survival curves of *glp-1(e2141)* mutants with postdevelopmental treatment of RNAi against OSCP/*atp-3*. Strains were developed at 25°C to induce sterility of *glp-1(e2141)* mutants. At young adulthood, nematodes were shifted to RNAi plates at 20°C for 48 hours and then moved to OP50 bacteria for the remainder of the lifespan. Representative curves were selected from three biological experiments. *glp-1(e2141)* CV RNAi (*n* = 137, median survival = 20 days), *glp-1(e2141) *OSCP/*atp-3* RNAi (*n* = 120, median survival = 23 days). *p = *0.0613 by Log Rank (Mantel–Cox) test

**Fig. 4:**
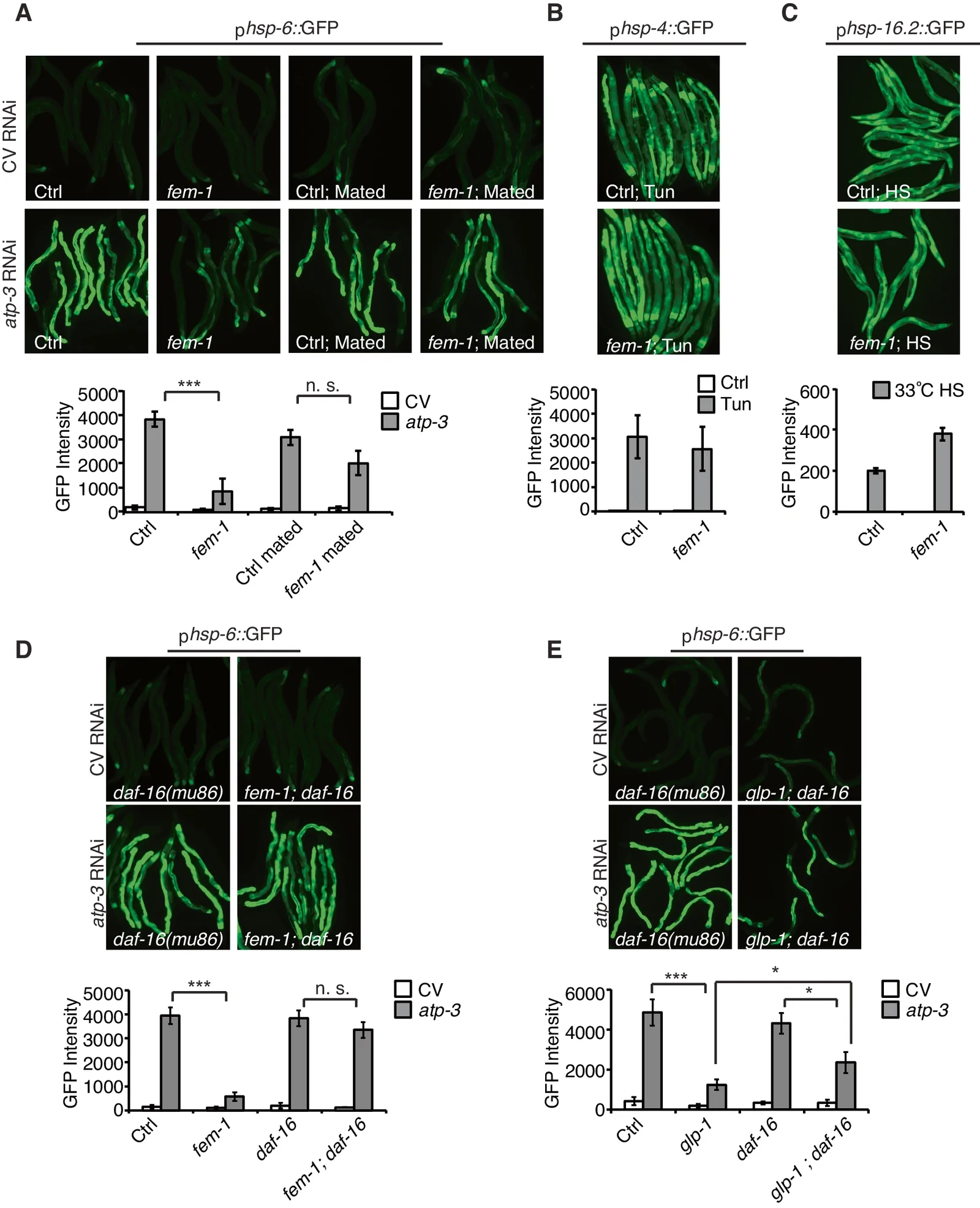
Late-stage reproductive signals are required for the intestinal UPR^mt^ activation, which is partially dependent on the transcription factor FOXO/*daf-16. ***A.** Top panels: Photomicrographs of p*hsp-6*::GFP or *fem-1*(*hc17*); p*hsp-6*::GFP reporter strains on bacteria expressing RNAi against OSCP/*atp-3*. Strains were developed at 25°C to induce sterility of *fem-1*(*hc17*) mutants. At young adulthood, nematodes were shifted to RNAi plates at 20°C for 48 hours. For matings, GFP reporter strain hermaphrodites at the L4 stage were introduced to young wild type males at a 1:4 ratio for 24 hours on RNAi plates. After 24 hours, hermaphrodites were transferred to fresh RNAi plates devoid of males. Mating was confirmed by the presence of eggs for each *fem-1*(*hc17*); p*hsp-6*::GFP hermaphrodite. Bottom panel: Quantification of GFP intensity. Data are the mean ± SEM of ≥ 45 animals combined from three biological experiments. ***p ≤ 0.0001 by Student’s *t*-test. n. s., non-significant. **B. **Top panels: Photomicrographs of p*hsp-4*::GFP or *fem-1*(*hc17*); p*hsp-4*::GFP reporter strains after treatment with 5μg/ml tunicamycin. Strains were developed at 25°C to induce sterility of *fem-1*(*hc17*) mutants. At young adulthood, nematodes were shifted to tunicamycin at 20°C for 48 hours. Bottom panel: Quantification of GFP intensity. No significant difference in GFP activation was observed between p*hsp-4*::GFP or *fem-1*(*hc17*); p*hsp-4*::GFP reporter strains after tunicamycin treatment. Data are the mean ± SEM of ≥ 45 animals combined from three biological experiments. **C. **Top panels: Photomicrographs of p*hsp-16.2*::GFP or *fem-1*(*hc17*); p*hsp-16.2*::GFP reporter strains after heat stress. Strains were developed at 25°C to induce sterility of *fem-1*(*hc17*) mutants. At young adulthood, nematodes were heat stressed for 1 hour at 33°C and GFP expression was analyzed approximately 5 hours later. Bottom panel: Quantification of GFP intensity. No significant difference in GFP activation was observed between p*hsp-4*::GFP or *fem-1*(*hc17*); p*hsp-4*::GFP reporter strains after heat stress. Data are the mean ± SEM of ≥ 45 animals combined from three biological experiments. **D**, **E.** Top panels: Photomicrographs of *daf-16(mu86*); p*hsp-6*::GFP or *fem-1*(*hc17*); *daf-16(mu86)*; p*hsp-6*::GFP (**D**) or *glp-1(e2141)*; *daf-16(mu86)*; p*hsp-6*::GFP (**E**) reporter strains on bacteria expressing RNAi against OSCP/*atp-3*. Strains were developed at 25°C to induce sterility of *fem-1*(*hc17*) and *glp-1(e2141)* mutants. At young adulthood, nematodes were shifted to RNAi plates at 20°C for 48 hours. Bottom panel: Quantification of GFP intensity for either *fem-1*(*hc17*); *daf-16*(*mu86*); p*hsp-6*::GFP or *glp-1*(*e2141*); *daf-16*(*mu86*); p*hsp-6*::GFP reporter strains compared to controls. Data are the mean ± SEM of ≥ 45 animals combined from three biological experiments. ***p ≤ 0.0001 by Student’s *t*-test. *p ≤ 0.01; n. s., non-significant

Here, we investigate how pharmacological or genetic inhibition of the germline affects mitochondrial protein homeostasis. The mitochondrial unfolded protein response (UPR^mt^) initiates a broad-range transcriptional response that aids in the refolding of mitochondrial matrix proteins, among other functions [4]. Boosting the UPR^mt^ is often associated with protection from mitochondrial insults and longevity [5]. However, the UPR^mt^ can also be a marker of deleterious processes, such as mitochondrial heteroplasmy [6], mitochondrial toxicity [7, 8], or the mitochondrial permeability transition pore (mPTP) [9]. We have observed that the intestine is the primary tissue of UPR^mt^ activation in *C. elegans* (Figs. 1A, B and 2A). The intestines of *C. elegans* run through the entirety of the nematode’s body. In addition to digestion, the *C. elegans* intestines carry out many functions similar to the liver, such as detoxification, immunity, and fat metabolism [10]. We report that FUdR treatment significantly inhibits the ability of *C. elegans* to mount the intestinal UPR^mt^, which is independent of its effects on the HSR (Fig. 1). Importantly, we additionally demonstrate that FUdR treatment impacts the trajectory of lifespans when in the presence of mitochondrial dysfunction, suggesting that the reproductive state can alter the rate of aging in the presence of mitochondrial dysfunction (Fig. 3). We additionally analyzed the UPR^mt^ in genetic germline mutants. Mutations in *glp-1* lead to premature entry of germline mitotic cells into meiosis [11]. The result is a small atrophied gonad but marked upregulation of somatic stress resistance, improved protein homeostasis, as well as significant lifespan extension, which are largely dependent on the upregulation of the transcription factor FOXO/*daf-16* [12, 13]*.* In addition to *glp-1* mutants, we utilized *fem-1* mutants, which are phenotypically female due to mutations that abolish sperm production [14]. We find that both *fem-1* and *glp-1* germline mutants inhibit the activation of the intestinal UPR^mt^ (Figs. 2 and 4), consistent with previous reports [15]. Strikingly, the intestinal UPR^mt^ is restored in *fem-1* mutants that have resumed reproduction due to mating with wild type males (Fig. 4A). Mechanistically, we find that loss of FOXO/*daf-16* completely restores the intestinal UPR^mt^ in *fem-1* mutants and partially in *glp-1* mutants (Fig. 4D). We conclude that the reproductive state of *C. elegans* informs mitochondrial protein homeostasis in distal tissues. The reproductive state of the germline may be a health indicator for distal tissues to modulate mitochondrial protein homeostasis.

## Results

To monitor the intestinal UPR^mt^, we utilized the transcriptional reporter strain, p*hsp-6*::GFP, which expresses GFP under the promoter of the mitochondrial Hsp70 molecular chaperone *hsp-6* [16]. After UPR^mt^ activation, GFP is robustly expressed throughout the intestines (Figs. 1A, B and 2A). We used two different methods to activate the UPR^mt^. Nematodes were treated with either 20mM manganese chloride (Mn) at the L4 stage [7] or RNAi of the F-ATP synthase subunit OSCP/*atp-3* at the young adult stage [9]. We found that the concomitant addition of either 10μg/ml (FUdR 10) or 100μg/ml FUdR (FUdR 100) robustly inhibited the activation of the intestinal UPR^mt^ by either Mn- or RNAi treatments (Fig. 1A, B) The effectiveness of FUdR as a DNA synthesis inhibitor was confirmed by the presence of unhatched embryonic eggs on plates (data not shown). To determine if FUdR treatment inhibited endogenous activation of the mitochondrial HSP-6 protein, we performed immunoblot analysis. The *C. elegans* HSP-6 protein is orthologous to and recognized by the mammalian GRP75/HSPA9 antibody. We found that 24 hour treatment with 100μg/ml FUdR reduced endogenous HSP-6 protein levels in nematodes that had been treated with OSCP/*atp-3* RNAi (Fig. 1C), consistent with the reporter strain results. We conclude that FUdR treatment inhibits the activation of the mitochondrial UPR^mt^ chaperone mtHsp70/HSP-6.

Given that FUdR treatment has previously been reported to activate the cytoplasmic heat shock response (HSR) [1–3], we sought to determine if FUdR may be affecting the UPR^mt^ due to its activation of the HSR. We crossed a strain harboring a mutation in the master HSR transcription factor HSF1 (*hsf-1(sy441))* to the UPR^mt^ p*hsp-6*::GFP reporter strain. We found that in the absence of deliberate UPR^mt^ activation, *hsf-1* mutants displayed a mildly elevated endogenous UPR^mt^ compared to non-mutant controls (Fig. 1B CV Ctrl vs.1D *hsf-1* CV Ctrl; **p ≤ 0.001). After RNAi of OSCP/*atp-3*, however, *hsf-1* mutants mounted a robust UPR^mt^ that was completely inhibited by FUdR treatments, indistinguishable from non-mutant controls (Fig. 1B, D). These findings suggest that the inhibition of the UPR^mt^ by FUdR is independent of its effects on the HSR. To determine the impact of FUdR treatment on other stress responses, we analyzed the endoplasmic reticulum UPR (UPR^ER^) using the transcriptional reporter strain, p*hsp-4*::GFP, which expresses GFP under the promoter of the ER Hsp70 molecular chaperone *hsp-4* [17]. We activated the UPR^ER^ via tunicamycin (Tun), a glycosylation inhibitor, in young adult nematodes and found that both low and high doses of FUdR significantly inhibited the UPR^ER^ (Fig. 1E). However, at its highest concentration, FUdR inhibited the UPR^mt^ by eightfold or more (Fig. 1B), while it only inhibited the UPR^ER^ by 2.8-fold (Fig. 1E). We conclude that FUdR treatment is specific in its ability to robustly inhibit activation of the intestinal UPR^mt^.

To determine if genetic inhibition of the germline recapitulated the pharmacological effects of FUdR, we tested nematodes with a temperature-sensitive mutation in the Notch-like receptor, *glp-1*, which inhibits germline stem cell proliferation and results in a nearly empty gonad. Similar to FUdR-treated nematodes, young adult *glp-1(e2141)* mutants inhibited the intestinal UPR^mt^ activation by sevenfold after RNAi of OSCP/*atp-3* (Fig. 2A). In contrast, young adult *glp-1* mutants were capable of robustly activating the UPR^ER^ as measured by the p*hsp-4*::GFP reporter strain (Fig. 2B) and the HSR as measured by the p*hsp-16.2*::GFP reporter strain (Fig. 2C), which expresses GFP under the promoter of the cytosolic molecular chaperone *hsp-16.2* [18]. We conclude that genetic inhibition of germline stem cell proliferation specifically and robustly inhibits activation of the intestinal UPR^mt^.

Next, we sought to determine whether FUdR treatment or *glp-1* mutations affect lifespan in the context of mitochondrial dysfunction. In the absence of FUdR, we have previously found that treatment with either Mn at the L4 stage or RNAi of OSCP/*atp-3* at young adulthood (postdevelopmental treatment) leads to lifespan shortening [7, 9] (Fig. 3A, B). The same lifespan treatments in the presence of FUdR, however, completely reversed the lifespan shortening so that the nematodes’ lifespans were indistinguishable from untreated control nematodes (Fig. 3A, B). Furthermore, we and others previously identified that RNAi of OSCP/*atp-3* beginning from eggs (developmental treatment) and continued throughout life leads to lifespan extension (Fig. 3C) [9, 19]. Remarkably, FUdR treatment also blocked the lifespan extension due to developmental RNAi treatment, even though FUdR treatment was not initiated until young adulthood (Fig. 3C). Next, we examined the effects of RNAi treatment of OSCP/*atp-3* on *glp-1* mutant lifespan. In contrast to wild type nematodes, the *glp-1* mutants did not display any significant lifespan shortening after postdevelopmental treatment with OSCP/*atp-3* RNAi (Fig. 3D). Thus, pharmacological or *glp-1* inhibition of the germline appears to guard *C. elegans* from the mitochondrial perturbations in the soma, regardless of whether the perturbations shorten or extend lifespan.

We next sought to determine mechanistically why inhibition of germline function would impact the ability of the intestines to mount the UPR^mt^. Recent studies have attributed embryonic signals as critical to somatic maintenance [20, 21]. To further explore what aspect of fertility is responsible for modulating the intestinal UPR^mt^, we tested nematodes with a temperature-sensitive mutation in the *fem-1* gene. The loss of *fem-1* function leads to the complete inhibition of sperm production, resulting in a feminized (female) gonad that develops quiescent oocytes. Like FUdR-treated and *glp-1* mutants nematodes, young adult *fem-1*(*hc17*) mutants did not robustly activate the intestinal UPR^mt^ after RNAi of OSCP/*atp-3* (Fig. 4A) but could activate the UPR^ER^ and the HSR (Fig. 4B, C). To determine if restoring fertility to *fem-1* mutants would also restore the intestinal UPR^mt^ activation, young adult *fem-1* mutants were mated with wild type males while exposed to RNAi of OSCP/*atp-3*. Mating was confirmed by the restoration of egg laying in the *fem-1* mutants (data not shown). Mated *fem-1* mutants did activate the intestinal UPR^mt^ after RNAi of OSCP/*atp-3* during young adulthood (Fig. 4A). Our findings with mated *fem-1* mutants suggest that the process of oocyte maturation, fertilization, and/or embryo deposition may modulate the intestinal UPR^mt^.

The FOXO/*daf-16* transcription factor is known to contribute to stress resistance, pathogen resistance, and longevity in *glp-1* mutants [13, 22]. Additionally, FOXO/*daf-16* is required for enhanced pathogen resistance in a variety of other sterile mutants, including FEM mutants, which can upregulate FOXO/*daf-16* downstream transcriptional targets [23]. We tested whether FOXO/*daf-16* activity contributed to intestinal UPR^mt^ regulation in *glp-1* and *fem-1* mutants. We found that introducing the *daf-16(mu86)* mutation completely restored the intestinal UPR^mt^ in *fem-1* mutants (Fig. 4D) and partially in the *glp-1* mutants (Fig. 4E). The *daf-16*(*mu86*) mutants alone could activate the intestinal UPR^mt^ similar to control levels (Fig. 4D, E). We conclude that FOXO/*daf-16* activity contributes to the suppression of the intestinal UPR^mt^, though other unknown mechanisms are also involved.

## Discussion

We find consistent evidence that inhibition of the germline by various means blunts the intestinal activation of the UPR^mt^. Our findings are consistent with the burst of recent research demonstrating that the germline is an essential tissue for UPR^mt^ signaling. Shen et al. (2024) demonstrated that the germline is required for neuronal to intestinal signaling [24]. Zhou et al. (2024) showed that embryo lystates transmit a hedgehog-like signal that modulates the somatic UPR^mt^ [21]. Similar to our findings, Charmpilas et al. (2024) demonstrated that FUdR-treated nematodes and *glp-1* and *fem-1* mutants do not activate the somatic UPR^mt^ and that mated *fem-1* mutants are able to restore the UPR^mt^ [15]. They further showed that young adult males were not capable of activating the UPR^mt^ [15]. This last finding, together with the finding that female nematodes (*fem-1* mutants) do not activate the UPR^mt^, suggests that signals from the combination of sperm and oocytes are essential to intestinal UPR^mt^ activation. The introduction of sperm, even defective sperm, leads to oocyte maturation, ovulation, fertilization, and embryo deposition [25]. Our previous work has shown that improved proteostasis by FUdR treatment requires sperm or sperm precursors, as feminized mutants are unaffected by FUdR [1]. Though it remains unknown whether FUdR treatment inhibits the intestinal UPR^mt^ by the same mechanism as *fem-1* mutants, it is possible that FUdR treatment compromises proper oocyte maturation or embryonic signaling, which would be consistent with the *fem-1* phenotype. The mechanism of FUdR has canonically been understood to be a thymidylate synthase (TS) inhibitor, which limits the formation of thymine and halts DNA synthesis. However, recent work has shown that the mechanism of FUdR in *C. elegans* after *E. coli* metabolism is not via TS inhibition but via the formation of a toxic monophosphate version of FUdR that leads to RNA metabolism defects among other effects [26–28]. How these effects may be influencing mitochondrial protein homeostasis or coinciding with the mechanism of *fem-1* mutations is unknown. Further studies are required to determine how FUdR treatment mechanistically inhibits the intestinal UPR^mt^ and whether this mechanism coincides with the germline mutant phenotypes.

Much research has been devoted to understanding how *glp-1* mutations extend lifespan and confer stress resistance [29]. Several transcription factors have been identified to mediate *glp-1* mutant longevity via inhibition of germline stem cell proliferation, with FOXO/*daf-16* being a central regulator [29]. In contrast, while sterility per se has not been regarded as a contributing factor to longevity [1, 30], there is evidence that sterile mutants, including *fem-1*(*hc17*) mutants, are resistant to pathogens in a FOXO/*daf-16*-dependent manner [22, 23]. Previous studies have also suggested that embryo signals may downregulate stress resistance and proteostasis, sometimes in a FOXO/*daf-16*-dependent manner [20, 23, 31]. We have identified that FOXO/*daf-16* activity fully suppresses the intestinal UPR^mt^ in *fem-1* mutants and partially in *glp-1* mutants. The partial suppression of the *glp-1* mutant phenotype by FOXO/*daf-16* may not be surprising given that its stress resistance and longevity can be mediated by a variety of factors, such as FXR/*daf-12*, HNF4/*nhr-80*, PPARα/*nhr-49*, TFEB/*hlh-30*, Nrf2a/*skn-1*, FOXA/*pha-4*, and HSF1/*hsf-1* [32]. It is possible that a combination of these transcription factors contributes to UPR^mt^ inhibition in *glp-1* mutants, which remains to be seen.

We additionally identify that loss of germline function affects lifespan trajectory in the presence of mitochondrial dysfunction, cautioning against using nematodes with altered germline function when studying the UPR^mt^ in *C. elegans*. Many studies continue to use FUdR while also studying proteostasis and mitochondrial dysfunction. Some studies have used *fem-1* mutants as a measure to avoid FUdR, but our results reveal that even *fem-1* mutations significantly alter the UPR^mt^ response. Nevertheless, avoiding the use of FUdR or sterility mutants presents a significant challenge to *C. elegans* researchers studying aging, since survival studies become significantly more labor intensive and it limits high-throughput methods. Adopting and refining methods that use fertile nematodes while removing unwanted progeny may be the optimal way forward to avoid confounding results [33].

## Methods

### Strains

Bristol N2 (wild type), SJ4005 (p*hsp-4*::GFP), and CL2070 (p*hsp-16.2*::GFP) nematodes were obtained from the *Caenorhabditis* Genetics Center (CGC, University of Minnesota) and cultured using standard conditions [34]. The following strains were generated in lab via genetic crosses: GL355 (BA17 *fem-1*(*hc17*) backcrossed 3× to N2), GL345 (CB4037 *glp-1*(*e2141*) backcrossed 3× to N2), GL347 (SJ4100 p*hsp-6*::GFP backcrossed 6× to N2), GL346 (*glp-1*(*e2141*); p*hsp-6*::GFP), GL364 (*fem-1*(*hc17*); p*hsp-6*::GFP), GL334 (*glp-1*(*e2141*); p*hsp-4*::GFP), GL376 (*fem-1*(*hc17*); p*hsp-4*::GFP), GL342 (*glp-1* (*e2141*); p*hsp-16.2*::GFP), GL397 (*fem-1*(*hc17*); p*hsp16.2*::GFP), GL367 (*daf-16*(*mu86*); p*hsp-6*::GFP), GL365 (*glp-1*(*e2141*); p*hsp-6*::GFP; *daf-16*(*mu86*)), GL369 (*fem-1*(*hc17*); *daf-16*(*mu86*); p*hsp-6*::GFP), SIA108 (*hsf-1*(*sy441*); p*hsp-6*::GFP).

### Nematode and bacterial culture conditions

Nematodes were maintained on nematode growth medium (NGM) plates. NGM plates were seeded with *Escherichia coli* OP50 obtained from CGC that was grown in LB at 37°C for 18 hours shaking at 225 rpm. Seeded plates were dried for 48 hours at room temperature before use. For RNAi experiments, HT115 (DE3) bacteria obtained from the Horizon Biosciences RNAi library were used [35, 36]. All RNAi clones were verified via sequencing. RNAi plates were prepared by cooling NGM to 55°C and supplementing with a final concentration of 50μg/ml carbenicillin and 1 mM isopropyl β-d-1-thiogalactopyranoside (IPTG). RNAi bacteria were inoculated with one colony of RNAi bacteria into LB with 50μg/ml carbenicillin and were grown shaking overnight for 18 hours at 37° at 225 rpm. RNAi cultures were seeded on RNAi plates and allowed to grow for 48 hours at room temperature. Plates were stored at 4 °C for no longer than 2 weeks.

For MnCl_2_ experiments, stock solutions of 3M manganese (II) chloride tetrahydrate (Sigma-Aldrich) were made in deionized/distilled water immediately before use. MnCl_2_ solution was added directly to NGM media at the indicated final concentrations before plates were poured After 24 hours, *E. coli* OP50 was spotted on MnCl_2_ NGM plates and spread by gentle swirling and allowed to grow for 24 hours at room temperature. Plates were stored at 4 °C for no longer than 2 weeks.

FUdR and tunicamycin were diluted in deionized/distilled and filtered water and spotted onto NGM plates previously seeded with OP50 *E. coli* or HT115 *E. coli* to a final concentration of 10μg/ml or 100μg/ml for FUdR or 5μg/ml for tunicamycin. Plates were dried at room temperature for 24 hours and subsequently stored at 4°C for no longer than 2 weeks.

### Lifespans

Day 1 adult nematodes were allowed to lay eggs for 2 hours on seeded NGM plates to obtain a synchronous aging population. Nematodes are transferred to freshly seeded bacterial plates every day for the first 7 days of adulthood and then as needed afterwards. Nematodes were scored as dead when they failed to respond to gentle prodding with a platinum wire. Nematodes that experienced matricide/bagging were censored. For developmental treatment lifespans, synchronized eggs were moved onto plates seeded with RNAi bacteria and maintained at 20°C for the entire lifespan. For postdevelopmental treatment lifespans, synchronized eggs were developed at 25°C on *E. coli* OP50 until nematodes were visibly past the L4 stage (loss of crescent) but not yet gravid (young adult stage), approximately 45 hours for wild type nematodes. At young adulthood, nematodes were shifted to 20°C and transferred to RNAi plates for 48 hours. After 48 hours, nematodes were transferred back onto regular NGM with *E. coli* OP50 for the remainder of their lifespan and remained at 20°C. When specified, 10 μg/ml FUdR was introduced at young adulthood for the remainder of the lifespan.

### Western blot

Approximately 40–45 adult nematodes were collected in S-basal buffer. Supernatant was removed, and nematodes were flash-frozen. Nematode pellets were resuspended in 2% SDS sample buffer with 2.5% β-mercaptoethanol, and samples were boiled for 10 minutes. Samples were subjected to SDS-PAGE using 4–12% SDS gels (Invitrogen) and transferred to PVDF membrane (Bio-Rad) using a semi-dry transfer blot apparatus. Membranes were blocked with 5% non-fat dry milk blocking solution; concentrations for antibodies were 1:1000 for primary antibodies and 1:2000 for secondary antibodies. We used GRP75 mouse mAb (D-9) (Santa Cruz Biotechnology sc-133137) and β-actin mouse mAb (Cell Signaling Technology 8H10D10).

### Microscopy

Nematodes were anesthetized with 2 -5mM levamisole and mounted on 2% agarose pads on glass slides. Fluorescence micrographs of GFP were taken using a Zeiss Axioscope 5 fluorescent compound microscope equipped with Zen microscopy imaging program. GFP expression was enhanced using the brightness/contrast tool in Adobe Photoshop. The same parameters were used for all images. GFP intensity of nematodes was quantified using ImageJ 1.54G. The “integrated density” of GFP expression and length of nematodes was measured using ImageJ tools. Integrated density value was normalized by number of nematodes and average length of nematodes. The final value is in arbitrary units.

### Statistics

Significance between control and experimental groups was determined by using a two-tailed Student’s *t*-test. Asterisks denote corresponding statistical significance: *p < 0.01; **p < 0.001; ***p < 0.0001. Error bars were generated using the standard error of the mean (SEM), typically from three or more pooled biological replicates. GraphPad Prism 10.6 was used to plot survival curves. Log rank (Mantel–Cox) test in Prism was used to determine significance between the control and experimental groups.

## Data Availability

Data generated or analyzed during this study are included in the manuscript. Worm strains generated from this study will be deposited and available via the Caenorhabditis Genetics Center.
